# Decontamination of High-Efficiency Mask Filters From Respiratory Pathogens Including SARS-CoV-2 by Non-thermal Plasma

**DOI:** 10.3389/fbioe.2022.815393

**Published:** 2022-02-14

**Authors:** Klára Obrová, Eva Vaňková, Michal Sláma, Jan Hodek, Josef Khun, Lucie Ulrychová, Filomena Nogueira, Triin Laos, Isabella Sponseiler, Petra Kašparová, Anna Machková, Jan Weber, Vladimír Scholtz, Thomas Lion

**Affiliations:** ^1^ St. Anna Children’s Cancer Research Institute (CCRI), Division Molecular Microbiology, Vienna, Austria; ^2^ Department of Physics and Measurements, University of Chemistry and Technology, Prague, Czech Republic; ^3^ Faculty of Science, University of Hradec Kralove, Hradec Králové, Czech Republic; ^4^ Institute of Organic Chemistry and Biochemistry of the Czech Academy of Sciences, Prague, Czech Republic; ^5^ Department of Genetics and Microbiology, Charles University, Faculty of Sciences, Prague, Czech Republic; ^6^ Department of Pediatrics, Medical University of Vienna, Vienna, Austria

**Keywords:** human respiratory viruses, influenza A, Rhinovirus, Adenovirus, *Pseudomonas aerguinosa*, particle filter, protective equipment, cold plasma

## Abstract

The current pandemic resulted in a rapidly increasing demand for personal protective equipment (PPE) initially leading to severe shortages of these items. Hence, during an unexpected and fast virus spread, the possibility of reusing highly efficient protective equipment could provide a viable solution for keeping both healthcare professionals and the general public equipped and protected. This requires an efficient decontamination technique that preserves functionality of the sensitive materials used for PPE production. Non-thermal plasma (NTP) is a decontamination technique with documented efficiency against select bacterial and fungal pathogens combined with low damage to exposed materials. We have investigated NTP for decontamination of high-efficiency P3 R filters from viral respiratory pathogens in comparison to other commonly used techniques. We show that NTP treatment completely inactivates SARS-CoV-2 and three other common human respiratory viruses including Influenza A, Rhinovirus and Adenovirus, revealing an efficiency comparable to 90°C dry heat or UVC light. Unlike some of the tested techniques (e.g., autoclaving), NTP neither influenced the filtering efficiency nor the microstructure of the filter. We demonstrate that NTP is a powerful and economic technology for efficient decontamination of protective filters and other sensitive materials from different respiratory pathogens.

## Introduction

Shortage of protective equipment during rapid epidemic spread of pathogens represents a recurring phenomenon (Hung, 2003; Beckman et al., 2013). The recent Covid-19 pandemic impressively revealed the magnitude of the problem if large parts of the world are affected within a short time. According to the World Health Organization (WHO), the demand for personal protective equipment (PPE) increased 100-fold during the spring of 2020, resulting in a dramatic shortage impossible to be counteracted by any achievable degree of pre-stocking. Therefore, shortages can readily occur in the initial phase of a global health challenge of this dimension, rendering research on emergency-reuse of PPE of critical importance (Godoy et al., 2020).

Guidelines for PPE application are typically standardized for one-time use, and highly efficient respiratory face masks (FFP2, FFP3, N95 or equivalent) should only be worn for a limited number of hours. Decontamination and reuse of PPE seem to be hampered primarily by damage leading to compromised fit or filtration efficiency (Bergman et al., 2012; Fisher and Shaffer, 2014; Godoy et al., 2020). The vulnerability of respiratory PPE materials implies the need for gentle procedures that maintain the required properties. Earlier data from Influenza epidemics show that PPE indeed becomes heavily contaminated with viruses, inevitably leading to hand contamination and further spread upon improper handling (Brady et al., 2017; Polkinghorne and Branley, 2020). To permit safe reuse of PPE, decontamination procedures need to inactivate all relevant pathogens including also bacteria introduced by breathing and manual handling by the wearer. Effective PPE decontamination therefore implies the need to cover a broader spectrum of pathogens beyond a particular virus of interest. Hence, if repeated use of PPE is intended, efficient decontamination procedures preserving the filtration properties are required. The use of non-thermal plasma (NTP) for treatment of PPE is an innovative strategy meeting these criteria. NTP is partially ionized gas inducible e.g., by electric discharge at ambient temperature and atmospheric pressure, where most of the kinetic energy is stored in free electrons (Ehlbeck et al., 2010; Scholtz et al., 2015). By collisions with ambient gas, these electrons generate excited or ionized particles and free radicals. In the air or similar environments containing oxygen, nitrogen and humidity, the most common particles include single delta oxygen, atomic oxygen and nitrogen, superoxide, ozone, hydrogen peroxide, nitric oxides and their intermediates as well as other components commonly referred to as reactive oxygen species (ROS) and reactive nitrogen species (RNS). Thanks to these particles, NTP possesses effective decontaminating properties, previously studied mainly in the context of antibacterial approaches (Ehlbeck et al., 2010; Scholtz et al., 2017; Julák et al., 2018; Scholtz et al., 2021) targeted towards food processing, treatment of agricultural products or various surfaces, including also medical applications (Graves, 2012; Ono, 2016; Dasan et al., 2017; Bourke et al., 2018; Misra et al., 2019; Scholtz et al., 2019). In recent years, the applicability of NTP for virus inactivation has gained increasing attention, including the possibility to tackle specific aspects of the current pandemic crisis (Bekeschus et al., 2020; Chen et al., 2020; Filipić et al., 2020). In addition to efficient decontamination properties, NTP offers a number of intriguing features including operation on demand (easy to turn on/off), readily transportable equipment, low maintenance requirements, performance at ambient temperature and atmospheric pressure, the possibility of dry conditions as well as low cost and easy manipulation. The gaseous nature of the active particles in NTP facilitates access to highly restricted spaces and penetration into micro- or nanoporous materials such as those used for PPE. It is estimated that around 10% of contaminating aerosols enter the inner layers of respiratory masks (Yi et al., 2005; Derraik et al., 2020), and are therefore shielded from decontamination by techniques that require direct access to the pathogens (e.g., UV light).

Various studies show that decontamination techniques differ substantially in their efficiency and inflicted damage, depending not only on the contaminating pathogen, but also on the PPE type and manufacturer (Derraik et al., 2020b; Boškoski et al., 2020; Polkinghorne and Branley, 2020). To obviate the need for testing a plethora of approved PPE types, we selected P3 R filters (Supplementary Figure S1) used as highly efficient protective membranes representing the most sensitive and therefore vulnerable part of respiratory PPE that can be attached to a variety of facemasks.

A number of reports highlight the poor transferability of laboratory decontamination results to real-world applications often revealing unexpected and dose-related damage to the treated materials by repeated cycles of decontamination, as shown e.g. for short-wavelength UV (UVC) light (Derraik et al., 2020b; Godoy et al., 2020; Polkinghorne and Branley, 2020). It is desirable therefore to employ long exposure in the experimental set-up to indirectly assess the impact of repeated decontamination cycles on the functionality of the exposed material. To cover viral pathogens displaying different properties and levels of resistance to chemico-physical challenges, we tested the inactivation of four important human respiratory viruses including SARS-CoV-2, Influenza A (IAV), human adenovirus (HAdV) and human rhinovirus (HRV). Additionally, the bacterium *Pseudomonas aeruginosa* was studied to compare the efficacy of this decontamination approach from bacterial and viral pathogens.

We report that NTP is able to reduce artificial contamination of P3 R filters by different respiratory viruses (SARS-CoV-2, IAV, HAdV and HRV) as well as *P. aeruginosa* below the detection limit of respective techniques. NTP therefore meets the requirements specified by the Food and Drug Administration (FDA) for a virucidal technique (Ludwig-Begall et al., 2020) for all pathogens tested. Unlike other decontamination techniques displaying similar efficacy, NTP did not cause any harm to the filter material, even after prolonged exposure for 24 h. The technique can therefore be exploited for decontamination of sensitive materials such as PPE devices, permitting their repeated use, particularly in times of shortage.

## Materials and Methods

### Artificial Contamination of P3 R Filters With Respiratory Pathogens

The following virus strains and cell lines were used: SARS-CoV-2 (hCoV-19/Czech Republic/NRL_6632_2/2020) isolated from a nasopharyngeal swab (Vaňková et al., 2020a) propagated in Vero E6 cells (ATCC CRL-1586) in DMEM with 2% FBS, Influenza A H1N1/California/07/2009 (Diagnostic Hybrids, Athens, OH, United States) propagated in MDCK (ATCC CCL-34) cells in Influenza growth medium consisting of DMEM 0.125% BSA; 10 mM HEPES; 2 μg/ml TPCK-Trypsin, human Adenovirus species C/type 2 (ATCC VR-846) propagated in A-549 cells (DSMZ ACC107) in DMEM with 10% FBS and Pen/Strep and human Rhinovirus species A/type 2 (ATCC VR-482) propagated in Hela Ohio cells (both a kind gift of Heinrich Kowalski) in DMEM with 10% FBS and Pen/Strep. Squares of 1 × 1 cm were cut from P3 R filters (ULPA (Ultra-Low Particulate Air) paper) and contaminated with 20 µL of virus suspension in culture medium displaying a median tissue culture infectious dose of 10^6^ infectious units (IU)/ml, applied in twenty droplets, each containing 1 µL solution.

For contamination with *P. aeruginosa*, sterile squares of 1 × 1 cm excised from P3 R filters (ULPA paper) were wetted with 10 × 1 µL PAO1 cell suspension adjusted to approximately 1 × 10^7^ CFU/ml. The samples were completely dried by maintenance for 1 h at room temperature.

### Technical Approaches Evaluated for Decontamination of P3 R Filters

The certified filtering efficiency of P3 filters (EN143: Respiratory protective devices—Particle filters) exceeds FFP3 mask performance (EN 149:2001 Respiratory protective devices—Filtering half masks to protect against particles), and P3 R filters can be attached to different half and full-face masks. The main difference between a filter and a respirator (N95, FFP2/3 masks or similar) is the material (ULPA or HEPA (High-Efficiency Particulate Air) fiberglass paper used in filters vs nanofiber or non-woven treated polypropylene) used in respirators. Another difference includes the filtration efficiency, which is generally higher in filters, but varies considerably. It is defined by EN: for example, the top FFP3 masks have a minimum efficiency around 99%, while the P3 filter has a minimum efficiency of 99.95% (sometimes 99.99+%). We selected this highly efficient filter used in PPE as model material for testing by artificial contamination and subsequent exposure to a number of decontamination techniques. The P3 R filters (Supplementary Figure S1), which contain randomly oriented silicate fibers with >18% alkaline oxide and alkali Earth oxide content, were placed in a removable plastic cartridge made from UV-stabilized polypropylene copolymer BE677AI containing folded ULPA fiberglass paper sealed with a hot-melt glue and nonwoven coarse particle filter. Exposure times used for the decontamination methods listed below were judiciously selected, employing greatest possible effort to mimic their use in real life. To reduce the probability of underestimating undesirable adverse effects on the materials treated, we selected extremely long exposure times (24 h) for the methods permitting such a length of exposure, and subsequently performed detailed studies assessing the filter microstructure and filtration efficiency. The following decontamination methods were applied to the complete P3 R filters (placed in a cartridge, Supplementary Figure S1):

*Boiling*—Samples were placed in boiling (100°C) tap water for 2 h and dried at room temperature (25°C) for 96 h. The 24-hour time point was omitted for this method due to major surface damage occurring already after 2 h.

*Ethanol*—Samples were submerged into 96% ethanol for 2 and 24 h and dried at room temperature (25°C) for 96 h.

*Dry heat*—Samples were placed in a laboratory oven and were tested under different conditions including 65, 90, 95 and 100°C for 15 min and 24 h.

*Autoclaving*—Samples were placed in an autoclave (Boeco Germany, BTE-23D) at 121°C and 0.1 MPa for 20 min.

*Peracetic acid vapors*—Samples were placed in a plastic box of 1 L volume containing peracetic acid (Persteril, OQEMA, Inc.) equilibrium vapors at 20°C for 15 min and 24 h.

*UVC irradiation*—Samples were placed between two sets of 3 UVC diodes (*λ* = 273 nm, power - 1–4 mW) at a distance of 7 cm and were irradiated from both sides for 2 and 24 h.

*Gamma irradiation*—Samples were placed in an electron accelerator and exposed to gamma irradiation at a dose of 25 kGy for 15 min.

*NTP exposure*—Samples were exposed to NTP generated by a device described in detail in our previous study (Lux et al., 2020) and schematically displayed in Supplementary Figure S2. The negative point-to-ring corona discharge burns at 7 kV and 150 µA. The device was enclosed in a plastic tube with a clear diameter of 4.6 cm and a length of 12 cm; the position of the electrode system was 2 cm from the tube outlet. The tube was mounted to the filter inlet, the gas of 1 L volume circulated in a loop. The filters were exposed for 15 min and 24 h in air atmosphere (denoted as air plasma) to generate a mixture of ROS and RNS or in oxygen atmosphere (denoted as oxygen plasma) to boost primarily ROS production.

Complete P3 R filters (Supplementary Figure S1) were exposed to the entire panel of decontamination methods. The filters were subsequently analyzed by electron microscopy and tested for maintenance of the filtering efficiency. Select methods were tested upon artificial contamination, as specified below.

### Disinfection of Virus-Contaminated P3 R Filters

Contaminated filters were exposed to the following methods: NTP (as described above, for 10, 30, 60, 90, 120 and 180 min), dry heat (15 min at 65 and 90°C in a thermoblock) and UVC (30 min using a standard biosafety cabinet lamp). Subsequently, residual virus was recovered from the filter surface using 180 µL PBS, the filter was washed thoroughly, the virus-containing solution was repeatedly re-distributed across the entire filter surface. Alternatively, 20 µL virus suspension (10^6^ IU/ml) was diluted to 180 µL with PBS and exposed to heat (15 min at 65 and 90°C in a thermoblock). To minimize the risk of bias, we employed a control including pipetting, drying, and recovery for every sample processed. The recovered suspension was used directly for the infection of permissive cells (specified above) to determine the infectious titer of each virus according to the respective protocols described below. SARS-CoV-2 was titrated by an immunofluorescence (IF) assay using a 1:2.5 serial dilution for infecting Vero-E6 cells (15,000 cells/well) in 96-well plates. Vero-E6 cells were incubated at 37°C in a 5% CO_2_ incubator for 72 h. After incubation, medium was removed, cells were washed once with PBS and fixed using 4% paraformaldehyde (PFA) for 15 min at room temperature (RT), washed 3× with PBS, permeabilized with 0.2% Triton-X100 for 5 min at RT, and incubated for 2 h with a 1:400 dilution of anti-SARS-CoV-2 antibody (mouse monoclonal nucleoprotein IgG, ProSci, CA, United States) at RT. Subsequently, cells were washed 3x with PBS, incubated for 1.5 h with a 1:250 dilution of Cy3-labeled donkey anti-mouse IgG (Jackson ImmunoResearch, Cambridgeshire, UK) at RT, and infected cells were visualized using a fluorescence microscope (Olympus IX 81, Germany). Influenza virus was titrated in focus forming assays using a 1:6 serial dilution. Virus was transferred onto MDCK cells (360,000 cells/well, 12-well plate), incubated for 1 h at 37°C in a 5% CO_2_ incubator. Then, virus was washed out and cells were overlayed with influenza growth medium containing 1% low-melting agarose. After 72 h incubation at 37°C in a 5% CO_2_ incubator, cells were washed once with PBS, fixed with 4% PFA for 15 min at RT, washed 3× with PBS, and incubated with blocking buffer (3% BSA, 0.3% Triton X-100 for 30 min at RT. After incubation, the cells were washed once with PBS and the developed foci were stained with a 1:1,000 dilution of anti-influenza antibody (mouse monoclonal nucleoprotein IgG, Merck, Darmstadt, Germany) in PBS with 3% BSA, 0.3% Triton X-100 for 1 h at RT, washed 3× with PBS, followed by incubation with a 1:250 dilution of Cy3-labeled donkey anti-mouse IgG (Jackson ImmunoResearch, Cambridgeshire, UK) in 3% BSA, 0.3% Triton X-100 for 1.5 h at RT. Foci were visualized using a fluorescence microscope (Olympus IX 81, Germany). The titer of Influenza virus was expressed as focus-forming units (FFU) per ml. Human Adenovirus was titrated by a TCID50 assay using a 1:10 serial dilution for infecting A549 cells in 96-well plates. A549 cells were incubated at 37°C in a CO_2_ incubator for 7 days, and the cytopathic effect (CPE) was quantified using the Crystal Violet assay, as described previously (Lee et al., 2015). Human Rhinovirus was titrated by a TCID50 assay using a 1:10 serial dilution for infecting Hela Ohio cells in 96-well plates. Hela Ohio cells were incubated at 37°C in a CO_2_ incubator for 5 days, and the CPE was quantified using the Crystal Violet assay, as described previously (Lee et al., 2015). Calculation of the respective infectious titers expressed in IU/ml was performed using the Spearman-Kärber method (Kärber, 1931; Spearman, 1908). Detection limits of individual assays based on sample sizes of 20 µL are indicated by IU, and revealed the following results: SARS-CoV-2: 32 IU, IAV: 62 FFU, HAdV: 76 IU, HRV: 76 IU. In addition, recovered HAdV and HRV genome copies were determined by quantitative PCR (qPCR), as described previously: HAdV (type C) (Lion et al., 2003; Watzinger et al., 2004), HRV (multiplex) (Deffernez et al., 2004). Recovered SARS-CoV-2 genome copies were determined by reverse-transcription quantitative PCR (RT-qPCR) using a commercial kit (gb Sarbeco E, cat. no. 3227-500, Generi Biotech, Hradec Kralove, Czech Republic) following the manufacturer’s instruction. Recovered Influenza genome copies were determined by RT-qPCR employing Luna Universal Probe One-Step RT-qPCR Kit (New England Biolabs, Ipswich, MA, United States) using 55°C for 10 min for cDNA preparation, followed by initial denaturation at 95°C for 1 min and 45 cycles of 95°C for 10 s and subsequent incubation at 60°C for 1 min (forward primer 5′GAC CRA TCC TGT CAC CTC TGA C3′, reverse primer 5′AGG GCA TTY TGG ACA AAK CGT CTA3′ and probe 5′-FAM-TGC AGT CCT CGC TCA CTG GGC ACG-BHQ1-3′) as published previously (Ngaosuwankul et al., 2010).

All data were generated in three biological replicates and statistically analyzed using Shapiro–Wilk test (to assess the data distribution). A *p*-value > 0.05 (in most cases very close to 1) indicated that the data follow a normal distribution. A *t*-test (two-sided test comparing treated sample and control sample at given timepoints) was therefore employed to determine the significance of differences. Results displaying *p*-values < 0.05 were considered significant and depicted as average ±standard error of the mean.

### Disinfection of P3 R Filters After Artificial Contamination With *P. aeruginosa*

The contaminated filters were exposed to following methods: NTP (as described above, for 10, 30, 60, 90 and 180 min), dry heat (65 and 90°C for 15 min in two different arrangements: 10 µL of inoculated dried bacterial suspension on filters or 10 µL of bacterial suspension in microtubes) and UVC (30 min using a standard biosafety cabinet lamp). Subsequently, residual bacterial cells—if present—were eluted from the filter surface by vortexing with 1 ml of sterile PBS in a microtube. The recovered suspension was used directly for inoculation of LB agar plates in ten-fold dilution steps. Inoculated plates were incubated at 37°C for 24 h before counting colony forming units (CFU). The experiment was carried out in three technical and three biological replicates. The results were averaged and expressed as log CFU/ml, and the difference between untreated and treated samples was evaluated by one-way analysis of variance (ANOVA) with a significance level of 0.05. To assess the metabolic activity of cells spotted on P3 R filter upon NTP treatment, a resazurin viability assay was used, as described previously (Vaňková et al., 2020b). Briefly, treated filters were submerged in resazurin/d-glucose solution, as described in the cited study, and cultured at 37°C using 150 rpm for approximately 1 h. The fluorescence intensity of created resorufin was measured at 545/575 nm. All data were generated in three biological replicates and statistically analyzed using Shapiro–Wilk test (to assess the data distribution). A *p*-value > 0.05 (in most cases very close to 1) indicated that the data follow a normal distribution. A one-way ANOVA test was therefore employed to determine the significance of differences. Results displaying *p*-values < 0.05 were considered significant and depicted as average ±standard error of the mean in relative percentages.

### Analysis of Filtering Efficiency by Aerosol Passage

Filter penetration was measured with an aerosol generator and a photometer (Lorenz Meβgerätebau FMP 03) with a differential pressure sensor, as described previously (Vaňková et al., 2020a). The device was certified as a test system according to the following standards: EN 143 (Respiratory protective devices—Particle filters—Requirements, testing, marking), and EN 149 (Respiratory protective devices—Filtering half masks to protect against particles—Requirements, testing, marking). The P3 R filter was mounted within the test system between the aerosol generator and the photometer. The aerosol generator produced a defined amount (6 mg/3 min ± 0.2 mg) of aerosolized paraffin oil, the test system passed it through the material, and the photometer situated on the other side of the sample measured the aerosol concentration, thereby indicating the retention efficiency (filter penetration). The particle size distribution was approximately 0.1–2 µm (geometric mean 0.44 µm), which is close to the most frequently observed penetrating particle size. The output of the aerosol generator was set to 150% with a flow of 95 L/min, an atomizer pressure of 5 bar and an oil temperature of 60°C. The test was performed for 3 min. Class P3 particle filters must meet the requirements for a maximum aerosol penetration of 0.05% (EN 143).

### Analysis of P3 R Filter Microstructure by Scanning Electron Microscopy

**FIGURE 1:**
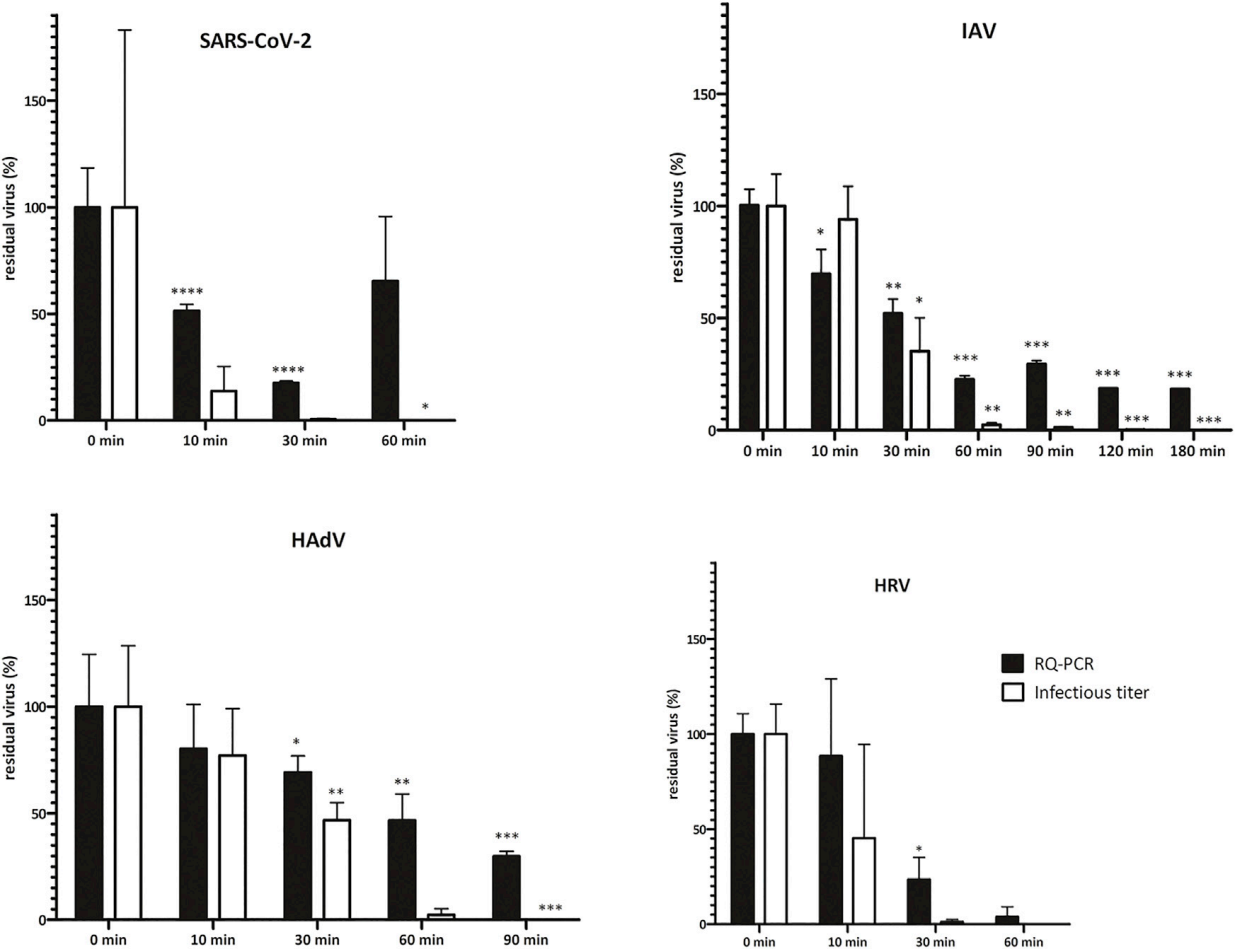
Virus infectivity upon NTP treatment is reduced more rapidly than genome copy numbers. Virus inoculum (20 μL, 10^6^ IU/ml) was applied onto P3 R filters, dried and exposed to NTP for the indicated times. Residual virus was recovered by PBS-mediated elution and titers were determined by TCID50 or IF assay (infectious titer) and qPCR (DNA/RNA genome copies). Data are plotted as % of untreated control samples (virus applied onto filters and kept in a laminar flow box for the respective times indicated on the abscissa); the mean of three biological replicates ±SEM is shown; *t*-test statistical analysis: * = *p* < 0.05, ** = *p* < 0.005, *** = *p* < 0.0005. Absolute values of virus titer are depicted in Supplementary Table S1. Virus infectivity (infectious titer) decreased with NTP exposure time and dropped below the detection limit of the assays employed after 60–180 min. The decrease in genome copy numbers (qPCR) reflecting physical integrity of the particles followed the same trend, albeit at a slower pace, indicating that infectivity was abolished before complete particle destruction was achieved. Fluctuations in test results reflect the complexity of the biological system. HAdV = human adenovirus, HRV = human Rhinovirus, IAV = Influenza A, SARS-CoV-2 = severe acute respiratory syndrome coronavirus 2, SEM = standard error of the mean.

The P3 R filters were exposed to all disinfection methods specified above and the fiber microstructure was subsequently analyzed using a scanning electron microscope (SEM) Nova NanoSEM 450 (Fei, United States). Approximately 1 × 1 cm pieces of both internal components of the treated P3 R filters (ULPA paper and coarse particle filter; Supplementary Figure S1) were completely air-dried and inspected using SEM with an LVD (low vacuum detector) at the following conditions: voltage of 10 kV, low vacuum, dwell time 20 μs and spot size 5.0 nm. Each sample was examined at 20 different locations under ×100 magnification and select representative images were scanned at a ×500 magnification. The obtained images were compared to open cartridges with treated P3 R filters in macroscopic pictures (Figure 1 and Supplementary Figure S1).

### Spectroscopic Analysis of the Hot-Melt Glue Holding the Filters

The possible influence of individual decontamination methods on the hot-melt glue holding the filter (Supplementary Figure S1) could manifest as oxidation or other changes affecting the composition (ratios) of involved elements. The element composition was measured by energy-dispersive X-ray spectroscopy (EDX). The measurement was performed by a Tescan Mira 3 LHM scanning electron microscope equipped with a Bruker XFlash 6I10 probe, which scanned the X-rays excited by the sample. The spectra of this radiation were evaluated with Bruker Quantax Esprit 2.0 software. The accelerating voltage was set to 10 keV. The scanned area of hot-melt glue sample was 0.5 × 0.5 mm^2^ under ×400 magnification. Each sample was measured at 5 different locations. The samples excised from the filter for subsequent analysis case had dimensions of ca. 4 mm width, 4 mm depth, and 1 mm height. Characterization of hot-melt glue was performed at the inner side of the sample, i.e. the element composition was measured inside the specimen volume.

## Results

### Impact of NTP and Other Decontamination Techniques on Viral Infectivity and Physical Integrity

**FIGURE 2:**
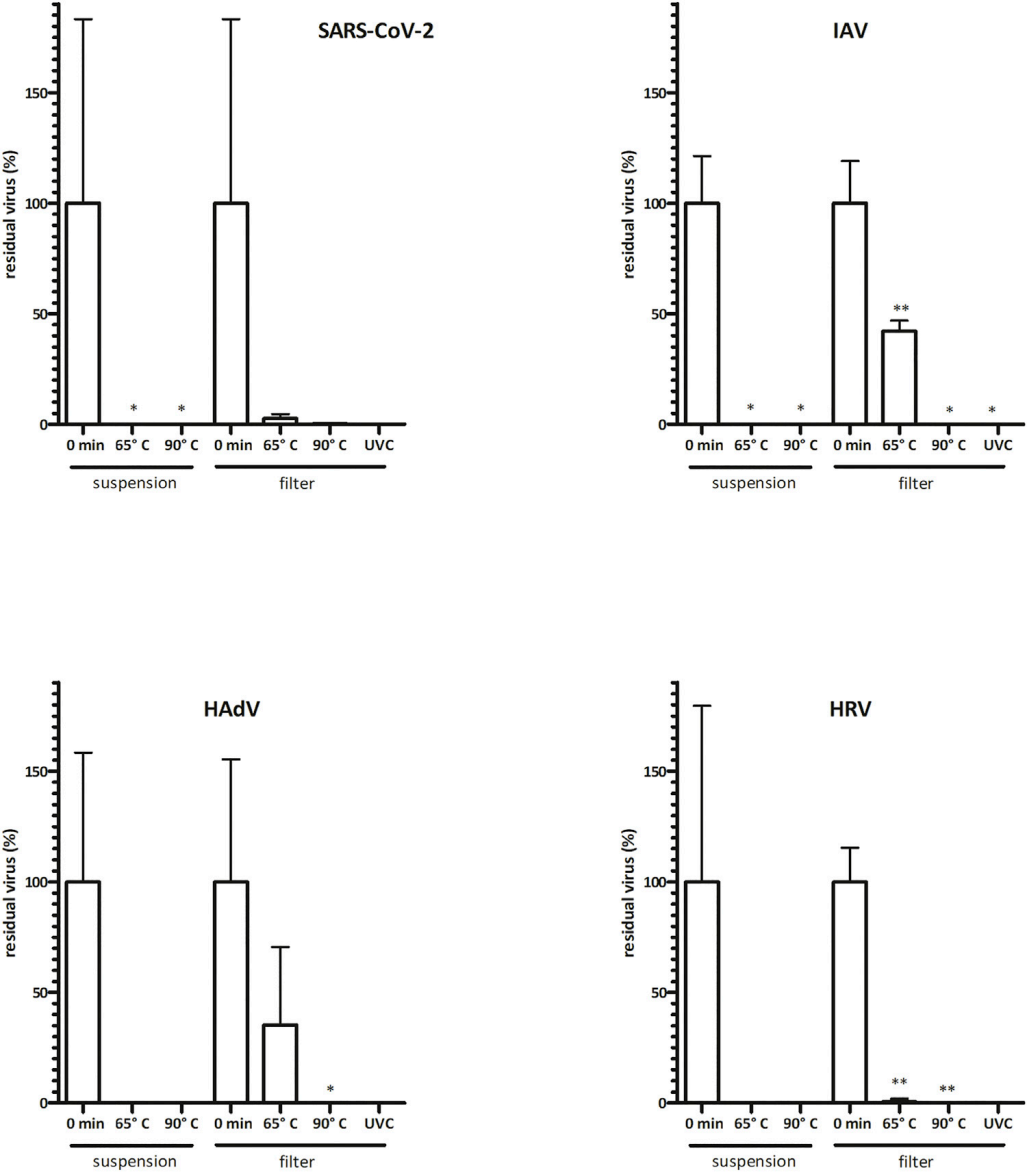
Virus infectivity is abolished upon commonly used disinfection methods. Virus inoculum (20 μL, 10^6^ IU/ml) was applied onto P3 R filters or was kept in suspension and exposed to dry heat (15 min) or UVC (30 min). Residual viruses were collected by washing with PBS, and titers were determined by TCID50 or IF assay (infectious titer). Data are plotted as % of untreated control samples (virus applied onto filters and kept in a laminar flow box for the respective times indicated on the abscissa); the mean of three biological replicates ±SEM is shown; *t*-test statistical analysis: * = *p* < 0.05, ** = *p* < 0.005. Absolute values of virus titer are indicated in Supplementary Table S1. Virus infectivity was only reduced upon thermal treatment at 65°C, revealing greater stability of virus particles treated on filters than those in suspension. Infectivity was completely abolished upon thermal treatment at 90°C or exposure to UVC. Fluctuations in test results reflect the complexity of the biological system. HAdV = human adenovirus, HRV = human Rhinovirus, IAV = Influenza A, SARS-CoV-2 = severe acute respiratory syndrome coronavirus 2, SEM = standard error of the mean.

To analyze the suitability of NTP-mediated decontamination, we artificially contaminated the P3 R filter with four common human respiratory viruses including SARS-CoV-2, IAV, HadV and HRV. Contaminated filters were exposed to NTP as well as other select decontamination techniques and residual viral infectivity and genome copies were determined and compared to control samples. NTP efficiently abolished the infectivity of SARS-CoV-2 and HRV after 60 min, HadV after 90 min, while IAV was fully inactivated after 180 min (Figure 1, Supplementary Table S1). The genome copy numbers, reflecting physical destruction of virus particles, decreased more slowly upon NTP exposure (Figure 1), dropping to 18–65% of residual particles at the time point of abolished infectivity. Dry heat treatment for 15 min at 65 and 90°C led to partial reduction of infectivity (Figure 2 and Supplementary Table S1). In all cases, virus particles applied onto P3 R filters were more stable than those undergoing thermal treatment in suspension, with IAV and HadV particles being most stable and maintaining 42.1 and 27.5% infectivity at 65°C, respectively. Exposure to UVC for 30 min completely inactivated all viruses (Figure 2, Supplementary Table S1).

### Efficacy of NTP and Other Decontamination Techniques on the Viability of *P. aeruginosa*

**FIGURE 3:**
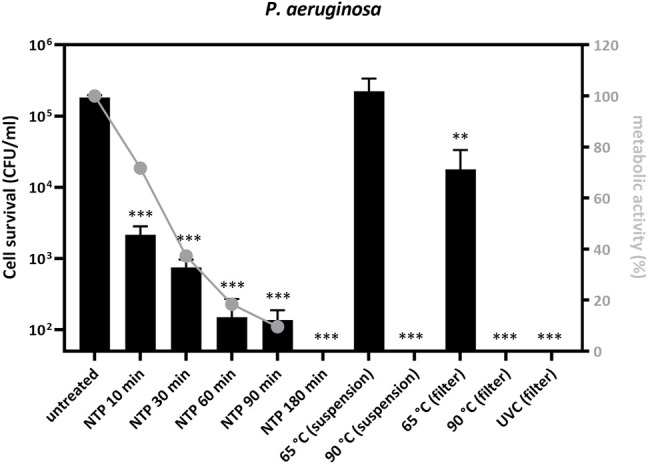
*Pseudomonas aeruginosa* PAO1 cell survival and metabolic activity decrease after treatment with NTP, dry heat or UVC. Bacterial inoculum (10 μL, 10^7^ CFU/ml) was applied onto P3 R filters and dried or treated as a suspension in microtubes, and subsequently exposed to NTP, dry heat or UVC for the indicated times. Filters with residual bacteria were immersed into 1 ml of PBS, vortexed and the entire suspension was transferred onto agar plates. Cell survival was determined and is indicated as log of colony forming units (log CFU) after 24 h of cultivation. Bacterial survival decreased with NTP exposure time and no colonies were observed after 180 min of NTP treatment. The decrease in metabolic activity followed the same trend, albeit at a slower pace, indicating that cell growth was impaired before the abolishment of metabolic processes. Bacterial survival was not changed upon thermal treatment at 65°C, revealing greater stability of cells treated in suspension than those on filters. Survival was completely abolished upon thermal treatment at 90°C or exposure to UVC. The mean of 3 technical and 3 biological replicates of bacterial cell survival reduction (expressed as log CFU) is depicted and the data were statistically analyzed using one-way ANOVA: ** = *p* < 0.005, *** = *p* < 0.0005. Metabolic activity of residual bacteria was determined using the resazurin viability assay, normalized to untreated controls, and plotted in relative %. NTP = non-thermal plasma, UVC = ultraviolet light C (laminar flow cabinet equipment).

To investigate P3 R filter decontamination from pathogens other than viruses, we additionally tested artificial contamination with the bacterium *P. aeruginosa* (Figure 3). NTP exposure decreased survival over time, resulting in a significant drop of CFU/ml by approximately 3 logs after 90 min of exposure (*p* = 0.0044) and complete abolishment of cell growth after 180 min (Figure 3). The assessment of metabolic activity (resazurin assay) revealed 90% inhibition following 90 min of treatment with NTP. The application of dry heat showed virtually no decontamination effect at 65°C for bacterial suspensions and only a half-log reduction in CFU/ml when testing contaminated filters. By contrast, treatment at 90°C completely abolished cell survival, both upon treatment of the bacteria in suspension and on the filters (*p* = 0.026). UVC was also proven to be very efficient, reducing cell survival to only 21.2 CFU/ml (*p* = 0.041).

### Effect of Decontamination Techniques on the Microstructure and Filtration Properties of P3 R Filters

**FIGURE 4:**
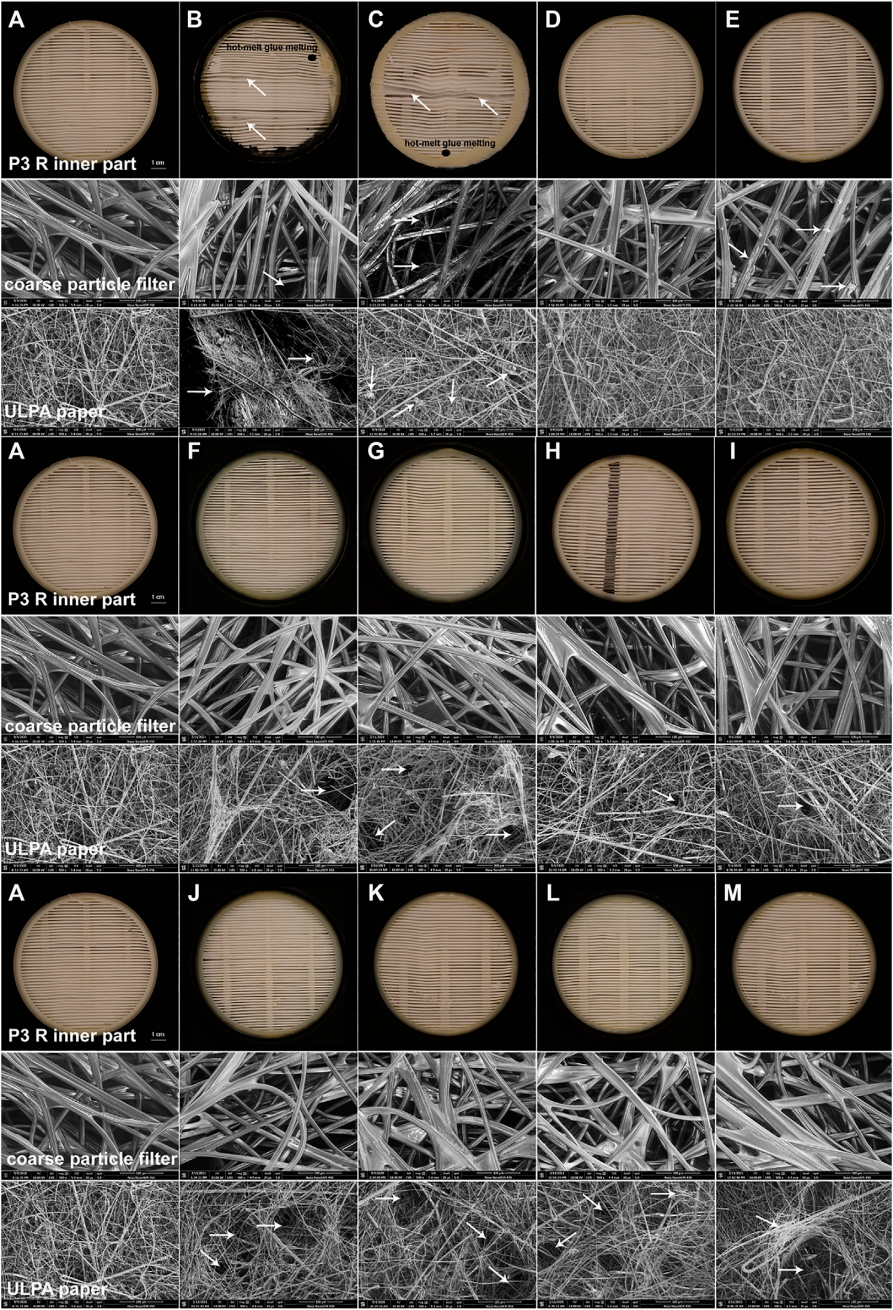
SEM images of damage detected in filter inserts of P3 R exposed to select disinfection methods. The complete P3 R filter in a cartridge was exposed to the decontamination techniques specified in the Methods section and photographed (inner part) or imaged using SEM at ×500 magnification (coarse particle filter, ULPA paper), focusing on the damaged areas typical for individual methods. The following conditions were investigated: **(A)** untreated control, **(B)** autoclaving at 121°C for 20 min, **(C)** boiling in tap water for 2 h, **(D)** soaking in ethanol for 24 h, **(E)** peracetic acid vapors (Persteril) for 24 h, **(F)** gamma irradiation for 15 min, **(G)** UVC irradiation for 24 h, **(H)** air plasma for 24 h, **(I)** oxygen plasma for 24 h, **(J)** dry heat at 65°C for 24 h, **(K)** dry heat at 90°C for 24 h, **(L)** dry heat at 95°C for 24 h, **(M)** dry heat at 100°C for 24 h. While most methods caused no or very limited damage, autoclaving and boiling clearly destroyed the filter, as documented by all testing approaches applied. Peracetic acid vapors formed a precipitate within coarse particle filters. The ULPA paper revealed small holes upon gamma irradiation, UVC or NTP treatments, and was substantially damaged upon all thermal treatments. White arrows indicate the damaged areas and black ellipses show places with hot-melt glue melting.

The greatest challenge for decontamination and reuse of protective respiratory masks is the sensitivity of the material to mechanical or chemical damage. Functionality of the material can be tracked by changes in filtration efficiency and microstructure. We exposed the P3 R filter to a number of decontamination techniques described in the Methods section for extended time periods, and carefully examined all detectable changes. After detailed examination by scanning electron microscopy, a number of smaller defects were detected in the microstructure (Figure 4A-M)—including damaged coarse particle filter fibers with precipitated material upon peracetic acid vapor treatment (Figure 4E), small holes in the ULPA paper fiber body upon gamma irradiation (Figure 4F), UVC (Figure 4G) and plasma treatments (Figures 4H,I), and substantial changes to the ULPA paper fiber body structure upon thermal treatment (Figure 4J-M). However, only autoclaving and boiling were associated with prominent damage to all parts of the filter, including the misalignment of coarse particle filter fibers after autoclaving (Figure 4B) and formation of small nodular structures (possibly broken and folded fibers) in the ULPA paper after boiling (Figure 4C). The pronounced microstructure damage by these two methods also translated into compromised filtration capacity failing the requirements of the EN143 norm (Table 1). All other treatments including NTP preserved the filtration capacity, although fluctuations in penetration between individual methods as well as individual filters were common (Supplementary Table S2). Another component of the filter cartridge is the sealing hot-melt glue (Supplementary Figure S1). Our results show that none of the methods changed its chemical composition (Supplementary Table S3), but melting upon thermal treatment was observed, which could lead to compromised protection of the filter during handling.

**TABLE 1:** Characteristics of P3 R filters upon application of different disinfection methods P3R filters in cartridges (Supplementary Figure S1) were exposed to different disinfection methods and the filtration efficiency was measured by aerosol passage according to the standards EN 143 and EN 149, as outlined in the Methods section.

Method		*t* (h)	T (°C)	Filter penetration before decontamination (%)	Filter penetration after decontamination (%)	Result	
Type/medium	Application					Applicability	Time to reuse
Autoclaving	Vapor heating	0.33	121	0.00037	39.11500	Inapplicable—does not meet EN 143 requirements	—
Tap water	Boiling	2	100	0.00031	Absolute	Inapplicable—does not meet EN 143 requirements	—
Ethanol	Chemical soaking	2	RT	0.00038	0.00206	Applicable	After 4 days
		24	RT	0.00035	0.00018	applicable	After 4 days
Peracetic acid (Persteril)	Vapor atmosphere	2	RT	0.00023	0.00021	Applicable	After a few hours
		24	RT	0.00028	0.00037	Applicable	After a few hours
Gamma irradiation	Physical disinfection	0.25	RT	0.00031	0.00017	Applicable	After a few minutes
UVC irradiation	Physical disinfection	0.25	RT	0.00019	0.00045	Applicable	Immediately
		24	RT	0.00001	0.00011	Applicable	Immediately
Air plasma (CAP)	Ionized gas	2	RT	0.00031	0.00131	Applicable	Immediately
		24	RT	0.00029	0.00012	Applicable	Immediately
Oxygen plasma	Ozone	2	RT	0.00033	0.00851	Applicable	After a few minutes
		24	RT	0.00041	0.00109	Applicable	After a few minutes
Dry heat	Indirect heating	0.25	65	0.00038	0.00065	Applicable	After a few minutes
		24	65	0.00032	0.00006	Applicable	After a few minutes
Dry heat	Indirect heating	0.25	90	0.00055	0.00007	Applicable	After a few minutes
		24	90	0.00024	0.00025	Applicable	After a few minutes
Dry heat	Indirect heating	0.25	95	0.00055	0.00064	Applicable	After a few minutes
		24	95	0.00089	0.00018	Applicable	After a few minutes
Dry heat	Indirect heating	0.25	100	0.00081	0.00011	Applicable	After a few minutes
		24	100	0.00051	0.00015	Applicable	After a few minutes

Differences in filtration efficiency (Δ filter penetration) before and after treatment were calculated from averaged values originating from 20 replicates, and the disinfection methods were assessed for their practical applicability. While most methods only had a minimal impact on the filtration efficiency, autoclaving and boiling impaired it beyond the lower limit of EN standards. Time to reuse = time needed for complete filter recovery, t = time of exposure in hours (h), T = temperature of exposure, p = pressure at exposure, RT = room temperature, atm = atmospheric pressure

## Discussion

The current pandemic and its challenges have emphasized the need to develop novel approaches to fighting the dissemination of respiratory pathogens. The shortage of PPE in the initial phase of the pandemic has highlighted the potential benefit of reusing highly effective facial masks upon decontamination from the pathogens. One of the promising technologies for inactivation of viruses and bacteria is NTP, which has been gaining increasing attention for this application. Thanks to its high efficiency even for treatment of challenging materials like absorptive or rough surfaces (Bekeschus et al., 2020; Chen et al., 2020), NTP is a candidate technique for decontamination of the vulnerable materials used for PPE production. In the current study, we tested NTP along with other commonly used techniques for decontamination of P3 R respiratory filters. P3R filters are used in combination with full masks or half-masks, which are typically made from resilient materials amenable to common decontamination techniques. Decontamination of masks suitable for filter insertion is also required and was studied previously (Vaňková et al., 2020a).

### Autoclaving Is Not Suitable for Decontamination of Respiratory Equipment

Autoclaving is a method of choice for sterilization of equipment in the laboratory and in medical settings. However, as shown by us and others, the method leads to profound damage of the sensitive respiratory PPE materials (Viscusi et al., 2009; Boškoski et al., 2020; Godoy et al., 2020; Rathnasinghe et al., 2020). We observed extensive damage to all parts of the P3 R filter (Figures 4B,C), which resulted in the loss of filtration efficiency (Table 1).

### Dry Heat-Mediated Inactivation Is Efficient, but Partially Destructive

Dry heat is a safe and readily available method for decontamination (Ludwig-Begall et al., 2020). We have tested four different dry heat temperatures including 65, 90, 95 and 100°C. Although the filtration efficiency of the filter remained unchanged during our testing (Table 1), we observed damage to the ULPA paper fiber body, substantially affecting its structure including the formation of holes (Figures 4L,M). Other parts of the P3 R filter also suffered from the thermal treatment, as indicated by melting of the hot-melt glue, which became more pronounced with increasing temperature. According to the safety data sheet, the plastic cartridge protecting the filter should not be exposed to temperatures above 50°C (or long-term above 35°C). We have nevertheless selected higher temperatures for inactivation testing in the current study based on our preliminary data on virus stability. While treatment at 65°C was not sufficient for complete inactivation of any of the viruses and caused either no or very minor damage to bacterial cells, exposure to 90°C completely inactivated all four viruses and *P. aeruginosa* (Figures 2, 3 and Supplementary Table S1). We also observed differences between direct application of dry heat to viral and bacterial suspensions as opposed to application to the filter. This observation emphasizes that conclusions cannot be made based on the general susceptibility of a given pathogen to a certain temperature but testing on the specific PPE material is required. Interestingly, while virus particles spotted onto filters were more resistant to heat than those in suspension, the trend was opposite for bacteria (Figures 2, 3). A risk of applying heat to complex structures such as folded P3 R filters is uneven heating due to delayed rise in temperature, leading to relatively long exposure time required for heating of the entire structure (Darmady et al., 1961). The temperature gradient across the filter structure can facilitate survival of pathogens in underexposed areas and accumulation of damage in overexposed ones.

### Chemical Methods Cannot Be Recommended for Decontamination of Respiratory Equipment

Chemical methods (e.g., treatment by ethanol or peracetic acid vapors) have been tested for decontamination of respiratory equipment, with somewhat controversial results. For example, hydrogen peroxide vapors are recommended by the Centers for Disease Control and Prevention (CDC) and FDA (Godoy et al., 2020) as an inexpensive method capable of inactivating porcine coronavirus (a surrogate for SARS-CoV-2) within a few minutes (Ludwig-Begall et al., 2020). However, detailed testing of virus infectivity upon treatment was hampered by the pronounced toxicity of residual hydroxide for the tissue culture needed for virus titer determination (Ludwig-Begall et al., 2020). As of February 2021, the European Union classifies hydrogen peroxide as an explosive precursor with strictly regulated sale (EU Regulation 2019/1148), thereby reducing the availability of this method. Toxicity as well as the risk for damaging the respiratory filter layers are relevant concerns for most chemical decontamination approaches (e.g. by ethanol and other organic solvents disrupting the static electricity necessary for respirator mask efficiency) (Viscusi et al., 2009; Derraik et al., 2020b; Polkinghorne and Branley, 2020; Su-Velez et al., 2020). We observed precipitated material and damaged microstructure upon peracetic acid treatment (Figure 4E). Both chemical methods tested required time for drying and ventilation of the filter before reuse (Table 1), although the filtration efficiency was not influenced. Based on our data and in the light of novel disinfection alternatives, the chemical decontamination approaches tested in the current study therefore cannot be recommended for routine PPE disinfection.

### Irradiation-Mediated Decontamination Is Gentle and Efficient Against all Respiratory Pathogens Tested

We tested two irradiation-based techniques including gamma irradiation and short-wavelength UV light (UVC). As expected, both techniques maintained the filtration efficiency of the filters (Table 1), and exposure to UVC was also efficient in inactivating all respiratory pathogens tested (Figures 2, 3 and Supplementary Table S1). This confirms that irradiation techniques can be regarded as methods of choice, as concluded by a number of studies (Derraik et al., 2020; Boškoski et al., 2020; Su-Velez et al., 2020; Huber et al., 2021). However, a drawback for broad applicability of gamma irradiation is the requirement of expensive equipment and expert operating personnel. By contrast, UVC is widely available, but its application requires direct exposure of the contaminated surface (Mills et al., 2018; Derraik et al., 2020b; Polkinghorne and Branley, 2020), and the inactivation of viruses and bacteria that penetrated deeper into the multi-layered structure of respiratory masks might be compromised. This indicates the need for thorough testing of individual types of PPE and individual pathogens (Derraik et al., 2020b; Boškoski et al., 2020; Polkinghorne and Branley, 2020). An additional concern associated with high-energy irradiation techniques is the cumulative damage to the weakest parts of respiratory equipment upon repeated exposure (Lindsley et al., 2015; Derraik et al., 2020). This notion is supported by the warning against UV exposure of the protective plastic cartridge of the P3 R filter on the safety data sheet, because the filter cartridge is made of a polypropylene copolymer with a relatively high degradation rate when exposed to UV light (Shyichuk et al., 2005). In line with this concern, we observed occasional occurrence of small holes formed upon irradiation in the ULPA paper fiber body (Figures 4F,G), which might represent initial steps of structural damage.

### Non-Thermal Plasma Is a Potent Decontamination Technique Requiring Further Optimization

The P3 R filters were exposed to two types of NTP based on air or oxygen as gas atmosphere, respectively. Both methods maintained the microstructure (Figures 4H,I) and filtration efficiency (Table 1) of the filter. In contrast to UVC, NTP is not hindered by shadowing and does not require direct exposure of the surface due to the nature of reactive particles in plasma (Ehlbeck et al., 2010; Julák et al., 2018). The energy of NTP particles is supposedly less damaging to the tissue microstructure than high-energy UVC or gamma irradiation. However, similarly to the irradiation techniques, we observed small holes in the ULPA paper fiber body (Figures 4H,I) that had no influence on the functionality (Table 1) but could represent incipient damage upon NTP treatment. At this point we would like to address the frequently asked question of the so-called “plasma dose”, which has plagued the field of plasma medicine for a long time. Unfortunately, it has not been possible so far to implement uniform approaches to possible mutual comparison of the biocidal effects of different plasma sources. The mixture of plasma-chemical reactions is very complex and the interaction of plasma with living organisms is even more complicated. However, a procedure has at least been proposed for an empirical comparison of the microbicidal activities of different plasma sources (Shaw et al., 2015). The suggestion has also been pursued by our group several years ago (Khun et al., 2017), but unfortunately this idea has not spread to the wider awareness of plasma physicists and this question therefore still remains unanswered. Hence, it is currently not pertinent to try introducing a generally applicable figure indicating the “plasma dose”.

NTP produced in air atmosphere efficiently abolished the infectivity of SARS-CoV-2, IAV, HAdV and HRV and the viability of *P. aeruginosa* (Figure 1, Figure 3 and Supplementary Table S1). As expected, individual pathogens had different degrees of resistance to NTP, with *P. aeruginosa* maintaining the viability for more than 2 h, and HAdV particles being physically the most resilient of the viruses investigated, probably due to its stable DNA genome and non-enveloped capsid (Figure 1). Intriguingly, IAV particles and their infectivity were maintained for over 120 min exposure to NTP, despite their presumably vulnerable enveloped particles and single-stranded viral RNA genome. The data therefore indicate the potential benefit of optimization towards shorter exposure times to improve practical applicability of the technology. Nevertheless, the current results of virus inactivation by NTP are encouraging and indicate that virus particles apparently lose infectivity prior to the occurrence of physical destruction (Figure 1). This is an expected result, because the viral surface structures required for successful infection are prone to damage faster than the genome, which is hidden inside and protected by the protein shell. The observation emphasizes the need for employing biological assays in the evaluation of disinfection techniques. We observed a similar trend for *P. aeruginosa*, where metabolic activity decreased more slowly than cell survival (Figure 3).

Virus infectious titers and genomes detected in individual replicates of samples and controls revealed some fluctuations, which limited the basis for statistical analysis, despite the presence of clear trends. This limitation was also reported by others (Rathnasinghe et al., 2020), and reflects the complexity of the test system with variable amounts of virus particles that become exposed to decontaminating treatment and are then recovered for subsequent analysis. The fact that the findings presented are the first data set generated with the non-thermal plasma system employed highlights the need for caution in the interpretation of results and further careful evaluation of the technology.

Investigation of the hot-melt glue component (Supplementary Figure S1) by EDX analysis to determine the percentage of oxygen and carbon content after different decontamination procedures revealed no significant differences (Supplementary Table S3), suggesting that the hot-melt glue composition was not affected. However, as discussed above, exposure to high temperatures led to different degrees of melting. Similarly, the plastic cartridge may be damaged upon repeated exposure to heat or UVC according to the manufacturer, and this observation was not made after NTP treatment in the current study, albeit not confirmed by repeated testing. In fact, we did not observe any changes to P3 R filter components upon NTP treatment, except for the occurrence of small holes in the ULPA filter matrix not affecting the filtering capacity. Nevertheless, additional analyses would be required to exclude the possibility of long-term damage. Heat inactivation at 90°C and exposure to UVC inactivated all viruses tested as well as *P. aeruginosa* in a shorter time than NTP, but both methods have potential disadvantages including greater damage to the filter and the difficulty of application to multilayered PPE due to their complex composition (Viscusi et al., 2009; Lindsley et al., 2015; Mills et al., 2018; Derraik et al., 2020; Polkinghorne and Branley, 2020; Su-Velez et al., 2020), which presents no obstacle to thorough penetration by NTP. Due to the gas-related nature of plasma, NTP can be readily applied to surface decontamination of various objects, including both individual and multiple pieces. During NTP application, it is important to ensure that the generated plasma reaches the entire targeted surface. This can be achieved by appropriate placement of individual plasma sources to channel the ion wind generated onto the target surface or to employ reinforced gas circulation. All respiratory pathogens treated by NTP revealed complete inactivation within the tested time frame of exposure, thus meeting the FDA requirements for a decontamination technique (Ludwig-Begall et al., 2020). Our earlier data on NTP efficiency against *P. aeruginosa* growing in the form of resistant biofilms suggest that the technology is also efficient under more challenging conditions than those tested in the current study (Paldrychová et al., 2019; Vaňková et al., 2019; Paldrychová et al., 2020). The main advantages of the NTP technology include its extremely easy implementation requiring a simple plug-in device with a metal electrode placed within a shell that can be produced very inexpensively in any desired shape by 3D printing. In this way, the device can be easily used in any given environment, and the time of exposure to NTP can be adjusted to any requirement for a particular level of decontamination. Given the flexibility of the NTP technology and its optimization potential, it is a promising novel approach for decontamination of PPE from a variety of respiratory pathogens. Moreover, the technology can also be employed for the decontamination of other sensitive materials and devices that cannot be exposed to heat, moisture, chemicals and others, such as sensitive electric devices and items made from paper or sensitive fabrics.

## Data Availability

The raw data supporting the conclusion of this article will be made available by the authors, without undue reservation.
