# Supplementary material for: Decontamination of High-Efficiency Mask Filters From Respiratory Pathogens Including SARS-CoV-2 by Non-thermal Plasma
Source: Front Bioeng Biotechnol. 2022 Feb 14;10:815393. doi: 10.3389/fbioe.2022.815393 (PMC8883054; doi:10.3389/fbioe.2022.815393)
Supplement: Supplementary file 1 [file DataSheet1.docx]

SUPPLEMENT


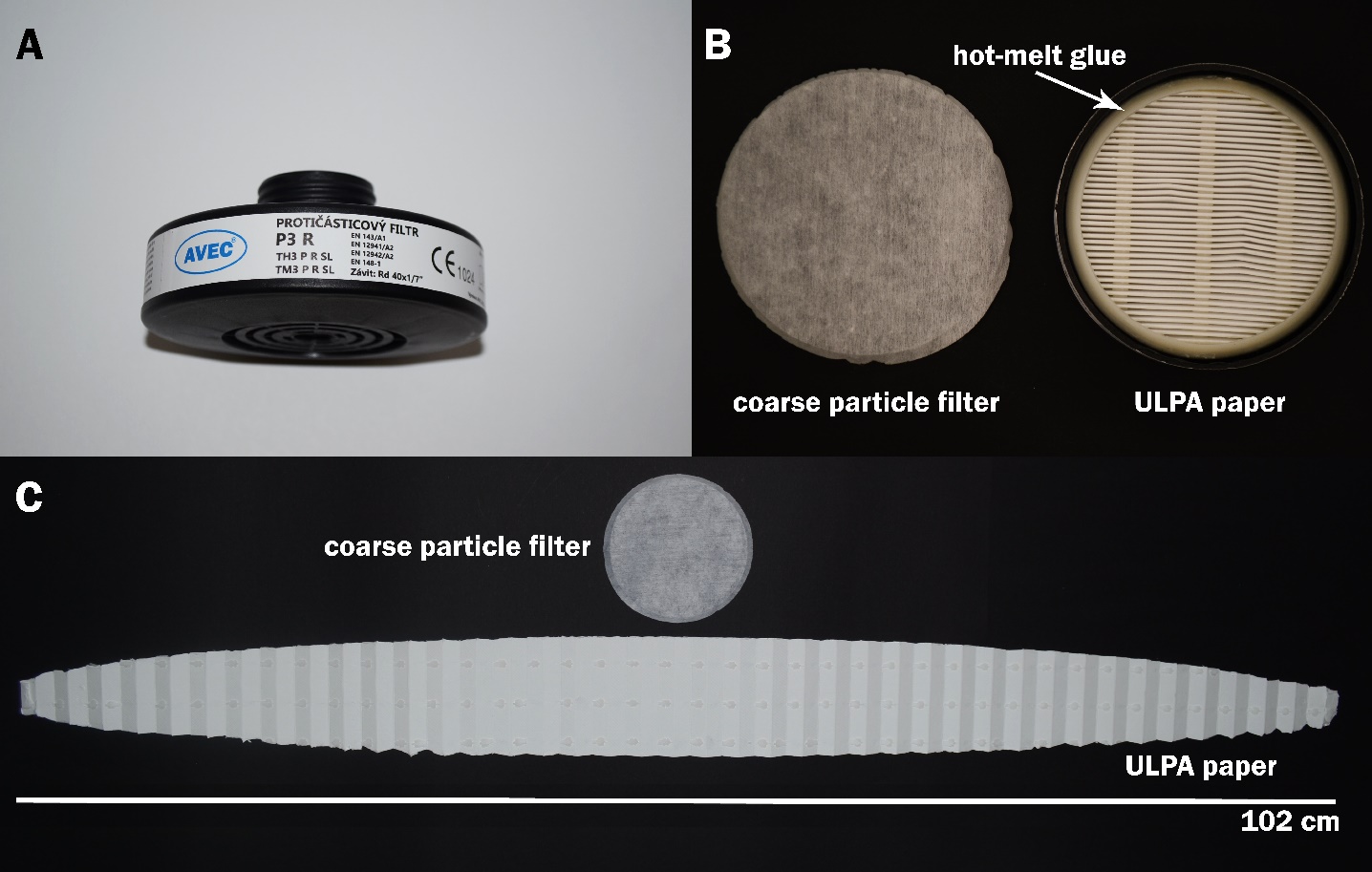


**Figure S1** P3 R filter composition. (A) Cartridge, (B) Filter insert (2 components: coarse particle filter and ULPA paper plus hot-melt glue sealing both components in the cartridge), (C) Size of coarse particle filter and ULPA paper in P3 R filters

**
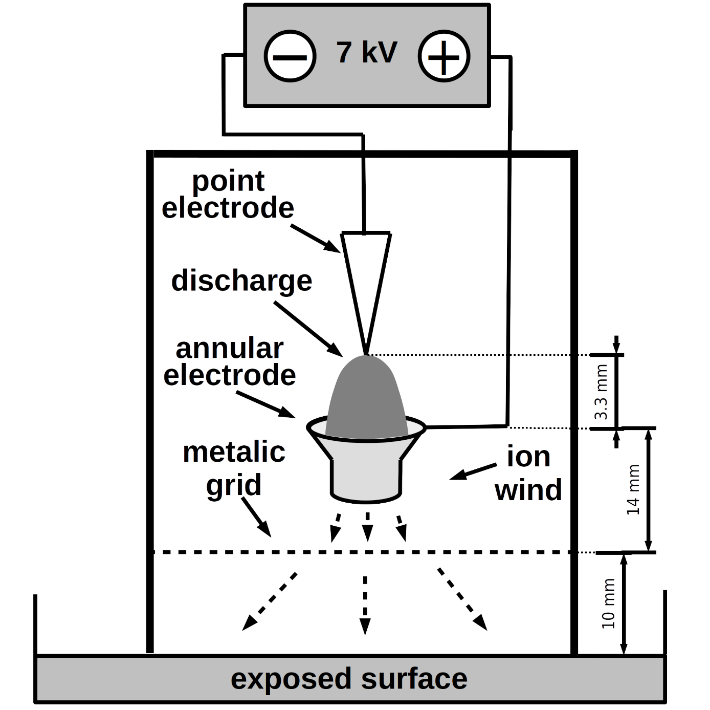
**

**Figure S2 Schematic representation of the plasma generator employed**

The set-up includes a closed chamber ensuring safe operational handling and higher concentration of the reactive species generated.

**Table S1 Elimination of virus infectivity upon treatment with NTP, dry heat or UVC.** Virus inoculum (20 µl, 10^6^ IU/ml) was applied onto P3R filters (filter) and dried or was treated in suspension within microtubes (suspension). The specimens were exposed to NTP, dry heat or UVC for the indicated duration. Residual virus was recovered by PBS-mediated elution and titers were determined by TCID50 (infectious titer). Detection limits of individual assays based on sample sizes of 20 µl are indicated by IU, and revealed the following results: SARS-CoV-2: 32 IU, IAV: 62 FFU, HAdV: 76 IU, HRV: 76 IU. The mean of 3 biological replicates ± SEM is shown. Relative reduction of virus titer after NTP treatment (% of control) are plotted in Figures 1 and 2. HAdV = human adenovirus, HRV = human Rhinovirus, IAV = Influenza A, SARS-CoV-2 = severe acute respiratory syndrome coronavirus 2, IU = infectious units, FFU = focus forming units, NTP = non-thermal plasma, UVC = ultraviolet light C (laminar flow cabinet equipment), RT = room temperature, SEM = standard error of the mean, n.a. = not applicable, n.d. = not done, compl. = complete reduction

| Method | Temperature  [°C] | Exposure time [minutes] | **SARS-CoV-2** | | **IAV** | | **HAdV** | | **HRV** | |
| --- | --- | --- | --- | --- | --- | --- | --- | --- | --- | --- |
|  |  |  | Infectious virus particles ± SEM [IU/ml] | Virus titer reduction [log] | Infectious virus particles ± SEM [FFU/ml] | Virus titer reduction [log] | Infectious virus particles ± SEM [IU/ml] | Virus titer reduction [log] | Infectious virus particles ± SEM [IU/ml] | Virus titer reduction [log] [%] |
| Control  (filter) | RT | 0 | 5.0·10^5^ ± 2.4·10^5^ | n.a. | 4.3·10^6^ ± 3.5·10^5^ | n.a. | 1.4·10^4^ ± 2.3·10^3^ | n.a. | 2.2·10^4^ ± 2.0·10^3^ | n.a. |
|  | RT | 10 | 5.8·10^5^ ± 1.9·10^5^ | n.a. | 4.3·10^6^ ± 3.5·10^5^ | n.a. | 5.7·10^3^ ± 9.9·10^2^ | n.a. | 1.4·10^4^ ± 6.2·10^3^ | n.a. |
|  | RT | 30 | 2.0·10^5^ ± 9.6·10^4^ | n.a. | 3.9·10^6^ ± 4.9·10^5^ | n.a. | 4.3·10^3^ ± 3.7·10^2^ | n.a. | 3.2·10^4^ ± 9.2·10^3^ | n.a. |
|  | RT | 60 | 1.2·10^5^ ± 3.1·10^4^ | n.a. | 2.3·10^6^ ± 3.4·10^5^ | n.a. | 2.4·10^3^ ± 1.1·10^3^ | n.a. | 2.2·10^4^ ± 9.9·10^3^ | n.a. |
|  | RT | 90 | n.d. | n.d. | 2.0·10^6^ ± 2.6·10^5^ | n.a. | 7.6·10^3^ ± 8.8·10^2^ | n.a. | 2.2·10^4^ ± 9.9·10^3^ | n.a. |
|  | RT | 120 | n.d. | n.d. | 1.7·10^6^ ± 6.4·10^4^ | n.a. | n.d. | n.d. | n.d. | n.d. |
|  | RT | 180 | n.d. | n.d. | 1.5·10^6^ ± 4.4·10^4^ | n.a. | n.d. | n.d. | n.d. | n.d. |
| NTP  (filter) | RT | 10 | 8.0·10^4^ ± 3.9·10^4^ | 0.86 | 4.1·10^6^ ± 3.7·10^5^ | 0.02 | 4.4·10^3^ ± 7.2·10^2^ | 0.11 | 6.1·10^3^ ± 3.8·10^3^ | 0.34 |
|  | RT | 30 | 1.0·10^3^ ± 5.3·10^2^ | 2.28 | 1.4·10^6^ ± 3.4·10^5^ | 0.44 | 2.0·10^3^ ± 2.0·10^2^ | 0.33 | 4.0·10^2^ ± 2.2·10^2^ | 1.91 |
|  | RT | 60 | 0 ± 0 | compl. | 5.5·10^4^ ± 1.1·10^4^ | 1.62 | 5.7·10^1^ ± 3.9·10^1^ | 1.63 | 0 ± 0 | compl. |
|  | RT | 90 | n.d. | n.d. | 2.4·10^4^ ± 3.2·10^3^ | 1.92 | 0 ± 0 | compl. | 3.4·10^1^ ± 3.4·10^1^ | 2.82 |
|  | RT | 120 | n.d. | n.d. | 4.8·10^3^ ± 1.1·10^3^ | 2.55 | n.d. | n.d. | n.d. | n.d. |
|  | RT | 180 | n.d. | n.d. | 0 ± 0 | compl. | n.d. | n.d. | n.d. | n.d. |
| Control (suspension) | RT | 15 | 5.8·10^5^ ± 1.9·10^5^ | n.a. | 4.6·10^6^ ± 5.6·10^5^ | n.a. | 5.1·10^5^ ± 3.2·10^5^ | n.a. | 8.5·10^4^ ± 7.6·10^4^ | n.a. |
| Dry heat (suspension) | 65 | 15 | 0 ± 0 | compl. | 0 ± 0 | compl. | 2.8·10^1^ ± 2.8·10^1^ | 4.26 | 0 ± 0 | compl. |
|  | 90 | 15 | 0 ± 0 | compl. | 0 ± 0 | compl. | 0 ± 0 | compl. | 0 ± 0 | compl. |
| Control (filter) | RT | 15 | 2.0·10^5^ ± 9.6·10^4^ | n.a. | 4.6·10^6^ ± 5.1·10^5^ | n.a. | 2.0·10^4^ ± 6.4·10^3^ | n.a. | 3.6·10^3^ ± 6.1·10^2^ | n.a. |
| Dry heat (filter) | 65 | 15 | 5.1·10^3^ ± 2.5·10^3^ | 1.59 | 1.9·10^6^ ± 1.3·10^5^ | 0.38 | 5.5·10^3^ ± 2.1·10^3^ | 0.56 | 2.5·10^1^ ± 2.5·10^1^ | 2.16 |
|  | 90 | 15 | 0 ± 0 | compl. | 0 ± 0 | compl. | 0 ± 0 | compl. | 0 ± 0 | compl. |
| Control (filter) | RT | 30 | 2.0·10^5^ ± 9.6·10^4^ | n.a. | 3.9·10^6^ ± 4.9·10^5^ | n.a. | 4.3·10^4^ ± 2.9·10^4^ | n.a. | 1.4·10^4^ ± 8.7·10^3^ | n.a. |
| UVC (filter) | RT | 30 | 0 ± 0 | compl. | 0 ± 0 | compl. | 0 ± 0 | compl. | 0 ± 0 | compl. |

**Table S2 Detailed** **characteristics of P3 R filters after treatment by different disinfection methods**

P3R filters in cartridges (Figure S1) were exposed to different disinfection methods and filtration efficiency was measured by aerosol passage according to the standards EN 143 and EN 149, as outlined in the Methods section. Differences in filtration efficiency (Δ filter penetration) before and after treatment were calculated from averaged values originating from 20 replicates and the disinfection methods were assessed for their practical applicability. Time to reuse = time needed for complete filter recovery, t = time of exposure in hours (h), T = temperature of exposure, p = pressure at exposure, RT = room temperature, atm = atmospheric pressure, nm = nanometers, kGy = kilo Gray

| **Method** | | **t (h)** | **T (°C)** | **filter penetration** before decontamination **(%)** | **filter penetration** after decontamination **(%)** | **Δ filter penetration** | **Δ filter penetration (%)** | **possibility of re-use** |
| --- | --- | --- | --- | --- | --- | --- | --- | --- |
| **type/medium** | **Application** |  |  |  |  |  |  |  |
| tap water | Boiling | 2 | 100 | 0.00031 | absolute | Absolute | absolute | impossible - does not meet EN 143 requirements |
| ethanol | chemical soaking | 2 | RT | 0.00038 | 0.00206 | -0.00168 | 542.11 | after 4 days |
|  |  | 24 | RT | 0.00035 | 0.00018 | 0.00017 | 51.43 | after 4 days |
| dry heat | indirect heating | 0.25 | 65 | 0.00038 | 0.00065 | -0.00027 | 171.05 | after a few minutes |
|  |  | 24 | 65 | 0.00032 | 0.00006 | 0.00026 | 18.75 | after a few minutes |
| dry heat | indirect heating | 0.25 | 90 | 0.00055 | 0.00007 | 0.00048 | 12.73 | after a few minutes |
|  |  | 24 | 90 | 0.00024 | 0.00025 | -0.00001 | 104.17 | after a few minutes |
| dry heat | indirect heating | 0.25 | 95 | 0.00055 | 0.00064 | -0.00009 | 116.36 | after a few minutes |
|  |  | 24 | 95 | 0.00089 | 0.00018 | 0.00071 | 20.22 | after a few minutes |
| dry heat | indirect heating | 0.25 | 100 | 0.00081 | 0.00011 | 0.00070 | 13.58 | after a few minutes |
|  |  | 24 | 100 | 0.00051 | 0.00015 | 0.00036 | 29.41 | after a few minutes |
| autoclaving | vapor heating | 0.33 | 121 | 0.00037 | 39.11500 | -39.11500 | 10571621.62 | impossible - does not meet EN 143 requirements |
| UVC irradiation | physical disinfection (273 nm) | 0.25 | RT | 0.00019 | 0.00045 | -0.00026 | 236.84 | immediately |
|  |  | 24 | RT | 0.00001 | 0.00011 | -0.0001 | 1100.00 | immediately |
| gamma irradiation | physical disinfection (25 kGy) | 0.25 | RT | 0.00031 | 0.00017 | 0.00014 | 54.84 | after a few minutes |
| peroxyacetic acid (Persteril) | vapor atmosphere | 2 | RT | 0.00023 | 0.00021 | 0.00002 | 91.30 | after a few hours |
|  |  | 24 | RT | 0.00028 | 0.00037 | -0.00009 | 132.14 | after a few hours |
| air plasma | ionized gas | 2 | RT | 0.00031 | 0.00131 | -0.001 | 422.58 | immediately |
|  |  | 24 | RT | 0.00029 | 0.00012 | 0.00017 | 41.38 | immediately |
| oxygen plasma | ozone | 2 | RT | 0.00033 | 0.00851 | -0.00818 | 2578.79 | after a few minutes |
|  |  | 24 | RT | 0.00041 | 0.00109 | -0.00068 | 265.85 | after a few minutes |

**Table S3 Elemental characterization of treated samples using energy dispersive X-ray spectroscopy (EDX).**

| **Sample treatment** | **Elemental contents [%]** | | | | | |
| --- | --- | --- | --- | --- | --- | --- |
|  | **C** | **N** | **O** | **Na** | **Al** | **Si** |
| reference | 80.1 ± 4.8 | 2.6 ± 1.1 | 15.8 ± 4.1 | 0.063 ± 0.031 | 0.216 ± 0.147 | 1.173 ± 0.967 |
| autoclaving | 85.2 ± 2.8 | 1.7 ± 1.1 | 12.4 ± 2.3 | 0.068 ± 0.034 | 0.129 ± 0.065 | 0.393 ± 0.198 |
| boiling (100 °C, 2 h) | 86.6 ± 1.7 | 1.5 ± 1.4 | 11.7 ± 1.0 | 0.017 ± 0.014 | 0.164 ± 0.078 | 0.093 ± 0.042 |
| peracetic acid (Persteril, 24 h) | 86.8 ± 1.1 | 2.1 ± 0.2 | 10.5 ± 1.0 | 0.003 ± 0.003 | 0.457 ± 0.052 | 0.144 ± 0.017 |
| air plasma (24 h) | 73.6 ± 1.5 | 2.1 ± 0.9 | 22.4 ± 1.2 | 0.000 ± 0.001 | 0.112 ± 0.053 | 1.704 ± 0.211 |
| oxygen plasma (24 h) | 83.7 ± 2.2 | 1.4 ± 1.5 | 13.6 ± 2.0 | 0.046 ± 0.013 | 0.504 ± 0.389 | 0.67 ± 0.419 |
| ethanol (24 h) | 90.8 ± 2.8 | 1.0 ± 1.4 | 7.7 ± 1.6 | 0.009 ± 0.011 | 0.357 ± 0.311 | 0.099 ± 0.057 |
| gamma rad. (25 kGy, 15 min) | 82.5 ± 1.4 | 3.6 ± 0.4 | 13.4 ± 1.3 | 0.067 ± 0.022 | 0.304 ± 0.074 | 0.111 ± 0.037 |
| UVC rad. (24 h) | 87.1 ± 1.8 | 1.6 ± 1.3 | 10.9 ± 0.7 | 0.032 ± 0.011 | 0.364 ± 0.077 | 0.078 ± 0.012 |
| dry heat (65 °C, 24 h) | 86.8 ± 1.0 | 1.9 ± 1.0 | 10.8 ± 1.2 | 0.030 ± 0.014 | 0.238 ± 0.097 | 0.165 ± 0.066 |
| dry heat (90 °C, 24 h) | 73.1 ± 2.4 | 3.4 ± 0.8 | 21.8 ± 2.1 | 0.113 ± 0.046 | 0.195 ± 0.129 | 1.357 ± 0.76 |
| dry heat (95 °C, 24 h) | 82.7 ± 3.4 | 2.1 ± 1.3 | 14.2 ± 2.5 | 0.232 ± 0.071 | 0.399 ± 0.391 | 0.414 ± 0.332 |
| dry heat (100 °C, 24 h) | 87.6 ± 3.0 | 1.4 ± 0.5 | 9.9 ± 3.1 | 0.106 ± 0.117 | 0.737 ± 0.448 | 0.261 ± 0.379 |
